# Ruminal Microbial Degradation of Individual Amino Acids from Heat-Treated Soyabean Meal and Corn Gluten Meal in Continuous Culture

**DOI:** 10.3390/ani12060688

**Published:** 2022-03-09

**Authors:** Silvia Gargallo, Alfred Ferret, Sergio Calsamiglia

**Affiliations:** Animal Nutrition and Welfare Service, Department of Animal and Food Sciences, Facultat de Veterinaria, Universitat Autònoma de Barcelona, 08193 Bellaterra, Spain; gargallosilvia@hotmail.com (S.G.); alfred.ferret@uab.cat (A.F.)

**Keywords:** soybean meal, corn glute meal, ruminal fermentation, amino acid

## Abstract

**Simple Summary:**

Dietary rumen undegradable protein provides almost half of the amino acids available for absorption. Current feeding systems assume that the amino acid profile of a protein reaching the small intestine is similar to that of the original protein. However, the results from the present experiment confirm that the degradability of individual amino acids from heat-processed soybean meal and corn gluten meal was different between protein sources and among amino acids within each protein source. The degradability of essential amino acids in general, and that of lysine in particular, was higher in both protein supplements, and the degradability of methionine was also higher in corn gluten meal compared with the average degradability of amino acids. Therefore, the flow of essential amino acids, lysin and methionine may be overestimated. Therefore, the results suggest the need to include the degradability of individual amino acids in precision feeding strategies.

**Abstract:**

Eight dual-flow continuous culture fermenters were used in three periods to study the effects of diets containing heat-treated soyabean meal (HSBM) or corn gluten meal (CGM) on ruminal microbial fermentation and the degradation of individual amino acids (AA). Treatments were a mix of non-protein nitrogen (N; urea and tryptone) that were progressively substituted (0, 33, 67 and 100%) for HSBM or CGM. Ruminal escape of AA was calculated with the slope ratio technique. Total volatile fatty acids (95.0 mM) and molar proportions (mol/100 mol) of acetate (59.3), propionate (21.8) and butyrate (10.5) were not affected by the treatments. As the level of HSBM or CGM increased, the concentration of ammonia-N and the degradation of protein decreased (*p* < 0.01), and the flows of nonammonia and dietary N increased (*p* < 0.01) quadratically. Compared with HSBM, CGM provided the highest flow (g/d) of total (20.6 vs. 18.3, *p* < 0.01), essential (9.04 vs. 8.25, *p* < 0.04) and nonessential (11.5 vs. 10.0, *p* < 0.01) AA, and increased linearly (*p* < 0.01) as the level of supplemental protein increased. Ruminal degradation of essential AA was higher (*p* < 0.04) than nonessential AA in CGM, but not in HSBM. Degradation of lysine was higher (*p* < 0.01) in both proteins, and degradation of methionine was higher in CGM. Ruminal degradation of individual AAs differ within and between protein sources and needs to be considered in precision feeding models.

## 1. Introduction

Current feeding systems use metabolizable protein as the unit to determine protein requirements and supplies [1,2,3,4]. Metabolizable protein reaching the small intestine is composed of microbial and dietary undegradable protein. However, ruminants have metabolic requirements for amino acids (AA) rather than protein. The supply of absorbable AA of dietary origin depends on the amount of feed AA escaping ruminal degradation and its intestinal digestibility. Current feeding systems calculate the AA supply to the small intestine using rumen undegradable protein (RUP) values and the AA profile of the original protein [1,2,3,5]. However, there is considerable evidence that the AA profile of ingested and undegradable feed protein varies among protein supplements [6,7,8]. Most available data on rumen degradability of individual AA within and between feeds were obtained using the in situ nylon bag technique [9,10,11]. However, there are some shortcomings in the methodology, as most estimates are obtained after 12 or 16 h of ruminal incubation and samples may be contaminated with amino acids from microbial origin, which may affect estimates of dietary AA supply [11,12]. The slope technique, originally proposed by Titgemeyer et al. [13], is a robust method to measure the rumen degradability of individual AA, but its application in vivo is complicated, labor intensive and expensive, and few data are available [13]. However, the methodology can be adapted to be used in in vitro dual-flow continuous culture rumen simulation systems, where microbial degradation of AA can be measured in conditions where type and amount of feed, dilution rates and pH are carefully controlled, and where the multiple interactions occurring during rumen fermentation are considered [14]. The hypothesis was that the increasing linear supply of heat-treated soyabean meal (HSBM) and corn gluten meal (CGM) will result in a linear increase in the flow of AA that can be attributed to the supply of these AA from their respective protein sources. The proportion of the marginal increase in the flow of each AA versus the supply of AA from each supplemental protein will represent the degree of rumen undegradability of each AA within each feed. This approach has already been used previously in a continuous culture system [14].

The objective of this study was to determine the effects of diets formulated to contain increasing amounts of HSBM or CGM on the ruminal escape of dietary AA in a dual-flow continuous culture system using the slope technique. 

## 2. Materials and Methods

### 2.1. Diets and Treatments

Eight isonitrogenous diets were formulated to meet or exceed NRC [2] nutrient recommendations for lactating dairy cows producing 40 kg/d of milk (Table 1). Diets were formulated with high crude protein (CP) content (22.0%) to provide: (1) sufficient rumen-degradable protein not to limit microbial protein synthesis in any diet, and (2) a sufficient amount of protein supplement (HSBM or CGM) to produce measurable effects on dietary AA flows. Nutrient composition and the AA profiles of HSBM and CGM are presented in Table 2. All diets were composed of a mix of a basal ingredient (71.2% of total DM) plus a protein supplement treatment (28.8% of total dry matter (DM)). Treatments consisted of a mix of feed-grade urea and tryptone (T9410, Sigma, St. Louis, MO, USA) plus a true protein supplement (HSBM or CGM) in the following proportions: 0% HSBM (HSBM-0), 33% HSBM (HSBM-33), 67% HSBM (HSBM-67), 100% HSBM (HSBM-100), 0% CGM (CGM-0), 33% CGM (CGM-33), 67% CGM (CGM-67) and 100% CGM (CGM-100). Corn starch (S4126, Sigma, St. Louis, MO, USA), limestone and wheat straw were used to adjust for total non-structural carbohydrates, ash and fiber content. All diets were ground at 1.5 mm screen (Hammer Mill Type O; *p*. Prat SA, Sabadell, Spain). The use of the slope technique in in vitro dual-flow continuous culture fermenters is an adaptation of the approach used by Titgemeyer et al. [13] to determine the degradability of individual AAs in vivo.

### 2.2. Continuous Culture System

Eight 1320 mL dual-flow continuous culture fermenters [15,16] were used in three replicated periods of eight days each. The fermenters were inoculated with rumen fluid strained through two layers of cheesecloth taken from two ruminally cannulated cows fed a 60 to 40 forage-to-concentrate diet. A total of 95 g of DM of each of the eight experimental diets was fed semicontinuously (0.66 g every 10 min) throughout the day to each fermenter to achieve steady-state conditions. The temperature was maintained at 39 °C and the pH controlled at 6.4 ± 0.05 by the infusion of 3 *N* HCl or 5 *N* NaOH. The fermentation conditions were monitored and controlled by a computer and a programmable linear controller, and were programmed with Lab View Software (FieldPoint, National Instruments, Austin, TX, USA). Anaerobic conditions were maintained by the infusion of N_2_ at a rate of 40 mL/min. Artificial saliva was continuously infused into the fermenters and contained 0.4 g/L of urea to simulate recycled nitrogen (N) [14]. The liquid and solid dilution rates were maintained at 10 and 5% h^−1^, respectively. A detailed description of the system was provided by Stern and Hoover [17].

### 2.3. Sample Collection

Each experimental period consisted of five days for adaptation and three days for sampling. During sampling days, the collection vessels were maintained at 4 °C to prevent microbial action. At the end of the day, the solid and liquid effluents were mixed and homogenized for 1 min, and a 500 mL sample was removed via aspiration. Upon the completion of each period, effluents from the three sampling days were composited and mixed within the fermenter. Subsamples were taken for total N, ammonia-N and volatile fatty acid (VFA) analyses. The remainder of the sample was lyophilized and ground through a 1 mm screen (Hammer Mill Type O; P. Prat SA, Sabadell, Spain). Dry samples were analyzed for DM, fiber, ash, purine bases and AA.

Solid (SAB) and liquid (LAB) associated bacteria were isolated from the fermenter flasks on the last day of each period. To obtain LAB, the fermenter contents were strained through two layers of cheesecloth. The solid fraction was washed with saline solution (8.5 g/L NaCl) in a three-to-one saline solution-to-solid fraction proportion to remove the residual bacteria of the liquid fraction [18], and the filtrate was added to the LAB preparation. The filtrate was centrifuged for 10 min at 1000× *g* to remove the feed particles. The obtained supernatant was centrifuged twice at 20,000× *g* for 20 min to isolate LAB. After the first centrifugation, the pellet was washed with saline solution, and after the second, the pellet was washed with distilled water to prevent the contamination of bacteria with ash. The final bacterial pellet was lyophilized.

The SAB were obtained by using a combination of several detachment procedures [19]. The fermenter solid residue was suspended in a saline solution with 1 g/L methylcellulose (proportion: three-to-one saline solution-to-solid fraction proportion), and incubated in a shaking water bath at 37 °C for 30 min to remove the solid attached bacteria [20]. After incubation, the samples were refrigerated for 24 h at 4 °C, and then shaken for one hour with marbles (30 2-mm and 15 4-mm diameter marbles) to dislodge the loosely attached bacteria. The solid fraction was strained through two layers of cheesecloth and washed three times with saline solution. The SAB pellet was obtained by differential centrifugation as in the LAB isolation procedure. The lyophilized LAB and SAB samples were composited within fermenters to obtain a single bacteria sample for each fermenter, and analyzed for DM, ash, N, and purine bases.

### 2.4. Chemical Analyses

Effluent DM was determined by lyophilizing 300 mL aliquots in duplicate with subsequent drying at 103 °C in a forced-air oven for 24 h. Dry matter content of diets and bacterial samples was determined by drying samples for 24 h in a 103 °C forced-air oven (ID 950.01; [21]). The dry samples were ashed overnight at 500 °C in a muffle furnace (ID 942.05; [21]). Total N in feed, effluents and bacterial samples was determined by the Kjeldahl method (ID 976.05; [21]). The neutral detergent fiber (aNDFom) and acid detergent fiber (ADF) concentrations in feed were determined sequentially using a thermostable amylase and sodium sulphite [22]. A 4 mL subsample of filtered fluid was acidified with 4 mL of 0.2 *N* HCl and frozen. Samples were centrifuged at 25,000× *g* for 20 min, and the supernatant was analyzed for ammonia-N by colorimetry [23]. Effluent and bacterial cells were analyzed for purine bases by HPLC using allopurinol as internal standard [24]. Samples for VFA were prepared using 4-methylvaleric acid as the internal standard [25] and analyzed by gas chromatography (Hewlett Packard, Palo Alto, CA). Effluent samples (5 mg) for AA analysis were hydrolyzed with 200 μL of 6 *N* HCl at 110 °C for 24 h in sealed, evacuated tubes. Mercaptoethanol was used as a reducing agent to prevent the oxidation of methionine residues during acid hydrolysis. Derivatization was conducted with 200 nmol per tube of dabsyl chloride at 70 °C for 12 min. Amino acid analysis was performed by reversed-phase HPLC ([26]; Beckman Instruments, Palo Alto, CA, USA). Norleucine was used as the internal standard and lysozyme was used as a standard protein of known AA profile to correct losses that may have occurred during the analytical process.

### 2.5. Calculations and Statistical Analyses

Flows and digestion of nutrients were calculated as described by Stern and Hoover [17]. Ruminal escape of individual AAs of HSBM and CGM were calculated as the ratio between the flows of each individual AA in the effluent (g/d) versus the AA intake (g/d) from the corresponding protein supplement. Data were analyzed with PROC MIXED of SAS (v 9.4, SAS Inst., Inc., Cary, NC, USA) with the model:

Y = Period + a + b1 × PS + b2 × LI + b3 × LI × PS + b4 × LI^2^ + b5 × LI^2^ × PS + b6 × LI^3^ + b7 × LI^3^ × PS. (1)

where period is the random effect, a is the intercept, and b1 to b7 are the coefficient for the linear, quadratic and cubic fixed effects of protein (P: HSBM and CGM), level of inclusion (LI: 0, 33, 67 and 100%) and their interactions. Starting with the full model, non-significant terms were deleted using the backward step-wise procedure until the highest-order term was significant at *p* ≤ 0.05. The 95% confidence interval of the de coefficient of the linear term of each AA was used to determine if the degradability of individual AAs was different from that of the total AA.

## 3. Results

Organic matter truly fermented was higher in HSBM vs. CGM and decreased linearly from 58.0 to 51.0, and from 55.1 to 48.5 % in HSBM and CGM, respectively (Table 3). Degradation of aNDFom was also higher in HSBM vs. CGM (34.0 vs. 29.0 %), but was not affected by the level of inclusion (Table 3). Concentrations of total VFA (average of 95.0 mM) and the molar proportions (mol/100 mol) of acetate (average of 59.3), propionate (average of 21.8) and butyrate (average of 10.5) were not affected by treatments (Table 3). Branched-chain VFAs (BCVFA) were affected by a protein source by the level of inclusion interaction, where the linear reduction was faster in HSBM (ranging from 5.0 to 3.13 mM) than in CGM (ranging from 4.9 to 4.15 mM).

The effect of treatments on N metabolism in fermenters is summarized in Table 4. Protein supplements had no effect in any of the measurements except for the efficiency of microbial protein synthesis. As the inclusion level of HSBM and CGM increased, ammonia N concentration showed a quadratic effect, but the coefficient for the quadratic term, although significant, was very small and the overall effect was almost linear (ammonia-N, mg/dl = 46.2 − 0.445x + 0.0012 x^2^). The flow of ammonia N followed a linear reduction as the level of protein supplements increased (Table 4). Nonammonia N flow showed a quadratic effect characterized by a saturation shape as the level of inclusion of proteins increased, and was due to the quadratic effect of dietary N flow. Changes in ammonia N concentrations and flows of ammonia and non-ammonia N (NAN) reflected the ruminal degradation of dietary proteins. Accordingly, the degradation of dietary protein decreased quadratically as the level of HSBM or CGM increased (Table 4). Microbial N flow showed a protein supplement by level of inclusion interaction, where it increased linearly in HSBM (from 1.03 to 1.27 g/d) but had no effect in CGM (average of 1.06 g/d; Table 4). The efficiency of microbial protein synthesis (g of N/kg of organic matter (OM) truly digested) of a protein supplement was obtained by measuring the level of inclusion interaction, where it increased linearly in HSBM (from 24.1 to 35.2) but had no effect in CGM (average of 28.9; Table 4). These differences were due to the greater efficiency of microbial protein synthesis in HSBM-67 and HSBM-100 compared with HSBM-0 and HSBM-33, resulting from the combination of an increase in bacterial N flow and the lower OM digestion observed in these treatments. 

The AA compositions of the two supplemental protein sources used in this study are presented in Table 2. Flows of total, essential and non-essential AA, Glu, Ser, Phe and Tyr were higher, and Lys was lower in CGM diets, and all increased linearly in both protein supplements as the level of supplemental protein increased (Table 5). Significant protein supplementation by level of inclusion interactions were observed only for the flows of Asp, Ala, Arg, Pro and Leu. These changes reflect CP degradation, the AA profile of each supplement, and the degradation rates of individual AAs. Models for the degradation of individual AAs were developed using the relationship between the amount of each AA supplemented in HSBM and CGM, and its flow, and are presented in Table 6. All models were linear, where the coefficient of the linear term reflects the degradability of each individual AA. Values less than 1 indicate that the AA was degraded more extensively than the total AA fraction. Values greater than 1 indicate that the AA was degraded less extensively than the total AA supplied by the supplement. The degradation of essential AA (EAA) was higher, and that of nonessential AA (NEAA) was lower in CGM. Similar trends were observed in HSBM, but differences were not significant. Degradation of Ile, Lys and Met were higher, and the degradation of Ala, Asp Glu, Gly, Pro and Tyr were lower than the average AA in CGM. Differences in the degradabilities of individual AAs in HSBM were lower, being significant only for Lys, which was more degraded, and Asp, Pro and Tyr, which were less degradable than the average AA in HSBM.

## 4. Discussion

Almost all research conducted to date investigating the rumen-degradability of individual AAs within feeds used the in situ technique [9,10,26], where most estimates were obtained after a single-point incubation, microbial colonization was not corrected in residual feed after incubation and the dynamic effects of the rumen were not considered, which may bias the estimates [12]. The slope technique is an alternative method to determine individual AA degradation. It is a robust experimental design that prevents some of the problems of the in situ technique. Diets were designed to achieve a similar flow of AA from the basal diet plus microbes, as previously suggested [13,14]. The hypothesis was that changes to the flow of AA within protein source out of fermenters could be attributed specifically to the increasing supply of AA from each level of protein supplement.

The linear reduction in OM truly fermented as the level of inclusion of protein supplements increased was expected due to the lower degradation of the protein fractions of HSBM and CGM compared with the extensive degradation of urea and tryptone in HSBM-0 and CGM-0 diets. Other authors also reported a decrease in OM degradation when rumen-protected protein sources were used instead of highly degradable protein supplements [14,27,28]. This linear reduction in OM degradation was parallel to the linear reduction in total VFA, although the differences did not reach significance. The lack of effect of the treatments on fiber degradation and the proportions of major individual VFAs indicated that the overall fermentation was similar among treatments, and that energy and protein availability did not limit microbial activity. The BCVFA (valerate, 2-methylbutyrate and isobutyrate) result from the deamination of the branched-chain AA (Leu, Ile and Val) and the changes observed reflect the content and degradation of these AAs in HSBM and CGM [28,29,30]. Within each supplement, the reduction in the concentration of BCVFA was also paralleled by a reduction in ammonia N concentration, and reflects the fact that BCVFA and ammonia N derive from the deamination of branched-chain AAs. 

The effects of treatments on N metabolism in the rumen are shown in Table 4. Ammonia N concentration showed a significant quadratic effect, although the numerical impact of the coefficient of the quadratic term was very small, and the practical effect was almost linear (ammonia-N, mg/dl = 46.2 − 0.445x + 0.0012 x^2^). The reduction in ammonia N concentration was expected, and in all cases was well above the 5 mg/dl suggested to maximize microbial growth, as expected [31]. In fact, the diets were designed so that N available for microbial growth would not be limiting. Similarly, other authors observed a reduction in ammonia N concentration when diets contained a rumen-protected protein source compared with the use of rumen-degradable protein in vivo [32] and in vitro [14,15,33]. The increase in NAN flow as the level of inclusion of HSBM and CGM increased was associated with a greater dietary N flow (Table 4). Clark et al. [34], using results of eight trials in which different sources of supplemental CP were fed, concluded that protein supplements with low ruminal degradability fed at high concentrations in the diet increased the passage of NAN to the small intestine compared with feeding soyabean meal (SBM), because of increased passage of dietary N. Changes in ammonia N concentration and flows of ammonia and NAN reflected ruminal degradation of dietary proteins, and agreed with previous in vivo and in vitro reports [13,15,29]. However, Clark et al. [34] reported that the increase in non-ammonia N flow was lower than expected based on the increase in the flow of dietary N, and was due to the parallel reduction in microbial N flow. In the present study, microbial N flow did not decrease as the level of supplemental protein increased. This was likely the result of the experimental design, where diets were formulated to provide, even at the highest inclusion level of RUP, sufficient degradable protein to maximize microbial protein synthesis. However, there was a significant protein source–level of protein inclusion interaction, where microbial N flow increased with an increasing level of inclusion in HSBM, but not in CGM. Because microbial N flow in CGM diets was constant and closer to the lower inclusion rates of the HSBM diets, the data suggest that microbial N synthesis was stimulated in the higher inclusion levels of HSBM. The reason is not clear, because ammonia N was sufficient to guarantee microbial growth and the amount of OM fermented in the rumen decreased in the diets with the high proportions of protein supplements. Titgemeyer et al. [13] suggested that if a significant percentage of dietary purines escaped ruminal degradation, bacterial N flows to the duodenum would be overestimated. However, McAllan and Smith [35] demonstrated that pure nucleic acids are rapidly degraded in the rumen. Moreover, Calsamiglia et al. [36] reported, using N^15^ as a marker, that dietary purines from HSBM and CGM were almost completely degraded by ruminal microbes in continuous culture, regardless of the total amount of purines in diets, and the escape of feed purine N seemed to be a minor factor affecting calculations of microbial nitrogen flow. This higher microbial N flow may compromise the underlying assumptions required for the calculations of the degradation of individual AAs from HSBM, which tend to be overestimated.

The degradation of dietary protein was not different between the HSBM and CGM diets, although it was numerically higher in the HSBM diets (41.2 vs. 37.3 %). Other authors found a trend for diets containing predominantly CGM to have lower protein degradation than diets containing treated SBM [2,15,30]. In fact, the NRC [2] recognizes a higher RUP level in CGM compared with HSBM. Within each protein supplement diet, there was a decrease in dietary protein degradation as the level of HSBM or CGM increased, according to the changes observed in the ammonia N concentration and the flows of ammonia and NAN.

The efficiency of microbial protein synthesis was within the ranges reported by Stern and Hoover [26]. There was a significant protein source–level of inclusion interaction, where it increased quadratically in HSBM, but remained constant in CGM. The greater efficiency of microbial protein synthesis of HSBM-67 and HSBM-100 resulted from the combination of an increase in bacterial N flow and the numerically lower OM digestion observed in these treatments. Coomer et al. [37] and Keery et al. [38] reported an increase in the efficiency of bacterial protein synthesis in steers fed diets supplemented with RUP compared with steers fed diets supplemented with untreated SBM. Cecava et al. [29] attributed changes in the efficiency of bacterial protein synthesis to differences in ammonia-N, AA and peptide availability for microbes. However, in the present experiment, the basal mix contained urea as a source of ammonia N, tryptone as a source of readily available peptides and AA, and the diets were fed semi-continuously every 10 min, providing N, AA and peptides on a constant basis throughout the day.

The increase in AA flows as the level of HSBM and CGM increased (Table 5) agrees with other studies, which reported that feeding low degradable protein supplements resulted in an increase in total AA flow [14,15,30]. The addition of CGM resulted in greater increases to flows of essential (EAA) and nonessential AAs (NEAA) compared with HSBM. Blake and Stern [30], in a continuous culture study, also reported higher EAA and NEAA flows with diets containing CGM than with diets supplemented with extruded whole soyabeans. Santos et al. [39] observed an increase in dietary AA flow when CGM was used as a source of supplemental protein, reflecting its higher degree of rumen undegradability [2]. Flows of Glu, Ser, Phe and Tyr were higher and those of Lys were lower for diets containing CGM compared with HSBM diets. However, there were no differences in Met flows between treatments. Blake and Stern [30] reported similar differences when comparing diets containing CGM or SBM. Similarly, Calsamiglia et al. [15] found that fermenters fed diets containing lignosulfonate-treated SBM had higher flows of Lys than fermenters receiving CGM-supplemented diets, but the differences in Met flows between both treatments were not significant. These results suggest that, although CGM provided large amounts of total AA, some potential limitations (low Lys) should be considered, and feeding combinations of protein supplements could improve the AA profile reaching the duodenum [13,15,29]. These results also suggest, that in spite of the higher content of Met in CGM diets, its flow was similar to HSBM, probably due to the extensive degradation of Met in CGM as compared with HSBM. 

All equations for the proportional flow of individual AAs from fermenters were linear. The coefficient of the linear term represents the ruminal escape of each AA from HSBM and CGM (Table 6). Overall, NEAA were less extensively degraded than EAA in CGM. Stern et al. [40] reported, in an in vivo study with cannulated cows, that the six most degradable AA in CGM was EAA. Particularly relevant was the higher rumen degradability of Lys in both protein supplements. The rumen degradability of Met was affected by an interaction (*p* < 0.06) with the protein supplement, where rumen degradability was higher in CGM, but not affected in HSBM. Isoleucine was also more degraded in CGM, but was not affected in HSBM. This result agrees with previous reports conducted in vivo with cannulated cows [40,41], in situ [7,42], and in vitro [15,30], which observed that Lys was one of the most degraded AAs among the EAAs. Conversely, Titgemeyer et al. [13] reported that the relative ruminal escape of Lys in CGM was lower than the total AA pool. Titgemeyer et al. [13] suggested that these differences may be explained by an increased Maillard product formation during processing that protected Lys from ruminal degradation. Some reports have shown Met to have higher degradability than total AA in several feeds [6,13,30]. However, data from other authors [7,43] have shown the degradation of Met to be dependent on the feedstuff. The degradation of branched-chain AAs was higher than total AA degradation in CGM, although some authors found these AAs to be more resistant to degradation in the rumen, depending on the protein source [7,12,13]. Alanine, Asp, Glu, Gly, Pro and Tyr were more degradable than the average total AA in CGM, and only Asp, Pro and Tyr in HSBM. Chalupa [44] found, in an in vitro system, that Tyr was degraded to a lesser extent when the fermentation system contained NEAA and EAA mixtures, and suggested that Tyr could be a degradative intermediate of Phe, therefore increasing the pool size of Tyr. Crooker et al. [43] reported that the proportion of Tyr increased or tended to increase as a result of ruminal exposure in 5 out of the 7 feeds tested in situ. Titgemeyer et al. [13] indicated that the rumen degradability of Tyr appears to be protein source-dependent, because it was degraded less than total AA in SBM and fish meal, but slightly more than total AA in CGM and blood meal. The lower ruminal degradation of Asp after the in situ ruminal exposure of several supplements was also reported previously [6,45]. Gonzalez et al. [12] suggested that hydrophobic non-polar AAs were less degradable in the rumen compared with hydrophilic polar AAs. Other factors, such as the solubility of the protein itself and the location of the protein within the structure of the feed or the protein, may also affect the rumen-degradability of individual AAs. 

If precision feeding of AAs is to be implemented in current feeding systems, differences within and among feeds of individual AA degradation in general, and EAA in particular, need to be considered. Rulquin and Vérité [46] stated that the modifications produced by rumen fermentation on dietary AA profiles could vary broadly according to feedstuffs and the level and degradability of the protein. However, because of methodological limitations and the small number of available data, these authors suggested using feed AA profiles as a first guide to estimate undegraded protein AA profiles. Currently, the recent version of INRA [3], the CNCPS system [5] and the NRC [2] used the AA profile of the original dietary proteins instead of that of the insoluble fraction. 

The results from the present experiment should be interpreted with caution, particularly in the HSBM treatment. The increasing flow of bacterial N in HSBM compromises the underlying assumption required to test the hypothesis and may overestimate the degradation rates of individual AA from HSBM. However, calculations of differences in the degradation of individual AAs within each protein supplement were performed relative to the degradation of total AAs, and this would reduce some of the bias. In contrast, the similar flow of microbial N in CGM validates the use of the slope methodology to calculate the differential degradability of AAs. 

This paper was designed as a robust alternative approach to evaluate the differential degradability of individual AAs within and between protein supplements. The results suggest that this occurs and affects particularly to EAA, including Lys and Met. If this is confirmed, flows of these AA would be overestimated in current feeding systems, and this effect may contribute to a limitation. The slope methodology is a robust design and the results were, in spite of the limitations of the methodology, consistent. Additional studies specifically designed to determine the differential degradability of individual AAs within and between proteins sources are necessary to advance theprecision feeding of AAs in dairy cattle diets because the magnitude of the differences is important. 

## 5. Conclusions 

Supplementing diets with increasing levels of HSBM or CGM reduces OM digestion without affecting NDF degradation, total VFA concentrations and the proportions of main VFA. The inclusion of these low-degradable protein sources increases the flow of dietary N and AAs. Diets with CGM provide the largest amount of total AA, EAA and NEAA. Microbial degradation of EAA is higher than NEAA in CGM. Microbial degradation of Lys is higher in HSBM and CGM, and that of Met is higher in CGM. The results from this study indicate that the microbial degradation of individual dietary AAs differs within and between protein supplements, and that the magnitude may be relevant. Further research is required to generate reliable and complete data in these and other feeds.

## Figures and Tables

**Table 1:** Ingredients and chemical composition of diets (% dry matter).

	Diet							
Item	HSBM-0	HSBM-33	HSBM-67	HSBM-100	CGM-0	CGM-33	CGM-67	CGM-100
Ingredients	71.2	71.2	71.2	71.2	71.2	71.2	71.2	71.2
Basal mix ^†^	-	9.5	19.0	28.4	-	-	-	-
HSBM ^‡^	-	-	-	-	-	7.14	14.2	21.2
CGM ^§^	3.38	2.17	1.12	-	3.43	2.90	1.86	0.91
Wheat straw	10.5	7.02	3.38	-	10.5	7.02	3.49	-
Tryptone	2.11	1.43	0.68	-	2.06	1.37	0.71	-
Urea	11.9	7.88	3.90	-	11.9	9.47	7.64	5.72
Starch	1.03	0.90	0.82	0.40	1.01	0.90	0.90	1.07
Limestone								
Analyses								
CP ^††^	21.7	22.0	21.8	22.1	21.7	21.8	21.9	21.8
NDF ^‡‡^	33.8	34.7	36.9	38.8	33.8	35.1	31.9	30.9
ADF ^§§^	20.2	19.9	20.6	21.4	20.2	21.0	18.9	18.4
NFC ^¥^	31.0	30.8	29.3	28.2	31.0	30.6	32.7	33.8

^†^ Contained (DM basis) alfalfa hay (19.7%), corn silage (18.2%), barley (16.5%), wheat straw (15.2%), vitamin-mineral premix (0.7%), tryptone (0.55%), salt (0.37%). ^‡^ HSBM = heat-treated soyabean meal. ^§^ CGM = corn gluten meal. ^††^ CP=crude protein. ^‡‡^ NDF=neutral detergent fiber. ^§§^ ADF=acid detergent fiber. ^¥^ NFC = non-fiber carbohydrates.

**Table 2:** Amino acid composition (g/100g of total AA measured) of heated soyabean meal (HSBM) and corn gluten meal (CGM).

Amino Acid	HSBM	CGM
Asp	6.8	1.61
Glu	13	8.37
Ser	6.51	5.47
Thr	5.1	4.11
Gly	5.11	3.35
Ala	5.69	8.66
Arg	8.32	3.53
Pro	5.76	10.6
Val	6.85	7.11
Met	1.53	2.9
Ile	7.11	7.31
Leu	10.3	22.9
Phe	6.28	8.55
Lys	7.23	2.51
His	2.93	1.6
Tyr	1.52	1.41
Essential ^†^	55.6	60.5
Nonessential ^‡^	44.4	39.5
Total	100	100

^†^ Includes Thr, Arg, Val, Met, Ile, Leu, Phe, Lys, His. ^‡^ Includes Ala, Asp, Glu, Gly, Pro, Ser, Tyr.

**Table 3:** Organic matter digestion and volatile fatty acid concentrations in continuous culture fermenters fed diets containing increasing levels of heated soyabean meal (HSBM) or corn gluten meal (CGM).

	HSBM				CGM					P < ^†^			
Item	0	33	67	100	0	33	67	100	SEM	P	LL	LQ	PxD
OMTF, % ^‡^	58.2	55.9	52.2	51	54.7	52.1	52.1	48.6	1.36	0.01	0.01	NS	NS
aNDFom fermented, % ^§^	35.2	34	33.5	33.8	25	30.3	31.6	29.2	2.5	0.05	NS	NS	NS
Total VFA, mM ^¥^	101	95.8	91.6	95	94.5	91.8	100	90.3	3.65	NS	NS	NS	NS
BCVFA, mM ^††^	5.21	4.16	3.19	3.23	4.71	4.29	4.11	3.92	0.26	NS	0.01	NS	0.02
Acetate, mol/100 mol	58.7	59.8	58.8	61.3	56.2	59.3	61.3	58.7	1.25	NS	NS	NS	NS
Propionate, mol/100 mol	22	21.1	22.2	21	23.1	21.4	21.6	21.9	0.98	NS	NS	NS	NS
Butyrate, mol/100 mol	9.8	10.5	11.2	10.6	11	10.4	9.2	11.3	0.6	NS	NS	NS	NS

^†^ None of the effects were cubic. S = effect of the type of supplement (HSBM or CGM); LL = linear effect of level of HSBM or CGM (0, 33, 67 or 100 % of supplemental protein). PxL = protein by level of inclusion interaction; NS = not significant, *p* > 0.10; ^‡^ OM=organic matter truly fermented; ^§^ aNDFom=neutral detergent fiber; ^¥^ VFA= volatile fatty acids; ^††^ BCVFA=branched-chain volatile fatty acids, including isobutyrate and isovalerate.

**Table 4:** Nitrogen metabolism in continuous culture fermenters fed diets containing increasing levels of heated soyabean meal (HSBM) or corn gluten meal (CGM).

	HSBM				CGM					P< ^†^			
Item	0	33	67	100	0	33	67	100	SEM	P	LL	LQ	PxD
Ammonia-N, mg/dl	46.3	32.7	23.7	15.6	47.6	31.9	23.2	12.5	1.13	NS	0.01	0.01	NS
Ammonia flow, g/d	1.45	1.03	0.75	0.5	1.51	1.02	0.73	0.4	0.03	NS	0.01	NS	NS
Nonammonia flow, g/d	2.46	2.98	3.44	3.59	2.53	3.09	3.33	3.58	0.05	NS	0.01	0.01	NS
Microbial flow, g/d	1.03	1	1.23	1.27	1.1	1.04	1.09	0.99	0.05	NS	NS	NS	0.01
Dietary flow, g/d	1.43	1.98	2.22	2.32	1.42	2.05	2.24	2.6	0.07	NS	0.01	0.01	NS
Protein degradation, %	57.2	41.6	33.9	32.1	57.2	38.7	33.4	23.3	2.02	NS	0.01	0.01	NS
EMPS ^‡^, g of N/kg OMTD	24.1	24.9	33.9	35.2	28.5	28.9	29.6	28.5	1.58	0.01	0.01	0.01	0.01

^†^ None of the effects were cubic. S = effect of the type of supplement (HSBM or CGM); LL = linear effect of level of HSBM or CGM (0, 33, 67 or 100 % of supplemental protein). PxL = protein by level of inclusion interaction. NS = not significant, *p* > 0.10; ^‡^ efficiency of microbial protein synthesis, in g of bacterial N per kg of organic matter truly digested (OMTD).

**Table 5:** Amino acid flow (g/d) from continuous culture fermenters fed diets containing increasing levels of heated soyabean meal (HSBM) or corn gluten meal (CGM).

	HSBM				CGM					P < ^†^			
Amino Acid	0	33	67	100	0	33	67	100	SEM	P	LL	LQ	PxD
Asp	1.53	1.91	2.44	2.71	1.53	1.99	1.96	2.18	0.12	NS	0.01	NS	0.01
Glu	1.97	2.37	3.15	3.5	1.97	3.47	3.59	4.66	0.181	0.01	0.01	NS	NS
Ser	0.75	0.89	1.15	1.27	0.75	1.13	1.2	1.43	0.05	0.01	0.01	NS	NS
Thr	0.77	0.89	1.12	1.2	0.77	1.05	1.07	1.18	0.037	NS	0.01	NS	NS
Gly	0.75	0.9	1.12	1.2	0.75	0.98	0.98	1.07	0.044	NS	0.01	NS	NS
Ala	1.2	1.43	1.55	1.69	1.2	1.93	1.97	2.6	0.093	NS	0.01	NS	0.01
Arg	0.65	0.89	1.18	1.25	0.65	0.88	0.89	0.99	0.041	NS	0.01	NS	0.03
Pro	0.71	0.8	0.95	1.04	0.71	1.25	1.3	1.7	0.073	NS	0.01	NS	0.03
Val	0.91	1.03	1.34	1.44	0.91	1.26	1.27	1.5	0.044	NS	0.01	NS	NS
Met	0.25	0.3	0.37	0.4	0.25	0.4	0.4	0.41	0.027	NS	0.01	NS	NS
Ile	0.75	0.88	1.21	1.31	0.75	1.09	1.1	1.34	0.048	NS	0.01	NS	NS
Leu	1.14	1.33	1.75	1.91	1.14	2.3	2.47	3.4	0.138	NS	0.01	NS	0.01
Phe	0.62	0.77	1.01	1.12	0.62	1.05	1.08	1.35	0.056	0.01	0.01	NS	NS
Lys	0.78	0.88	1.15	1.18	0.78	0.93	0.85	0.83	0.032	0.01	0.01	NS	NS
His	0.2	0.26	0.39	0.39	0.2	0.33	0.32	0.41	0.016	NS	0.01	NS	NS
Tyr	0.57	0.67	0.89	0.87	0.57	0.95	0.96	1.24	0.044	0.01	0.01	NS	NS
Essential ^‡^	6.06	7.24	9.51	10.21	6.06	9.28	9.44	11.4	0.369	0.04	0.01	NS	NS
Nonessential ^§^	7.48	8.97	11.26	12.29	7.48	11.7	11.96	14.88	0.526	0.01	0.01	NS	NS
Total	13.54	16.21	20.77	22.49	13.54	20.98	21.4	26.28	0.873	0.01	0.01	NS	NS

^†^ None of the effects were cubic. S = effect of the type of supplement (HSBM or CGM); LL = linear effect of level of HSBM or CGM (0, 33, 67 or 100 % of supplemental protein). PxL = protein by level of inclusion interaction. NS = not significant, *p* > 0.10; ^‡^ includes Thr, Arg, Val, Met, Ile, Leu, Phe, Lys, His; ^§^ includes Ala, Asp, Glu, Gly, Pro, Ser, Tyr.

**Table 6:** Prediction equation of the flow of individual AAs (mg) from the dual-flow continuous culture fed diets with increasing amounts of heat-treated soybean meal (HSBM) and corn gluten meal (CGM). The linear term (x) is the amount of AA (mg) supplied from the protein supplement, and its coefficient represents the proportion of the individual AA from the protein supplement escaping from ruminal degradation.

Amino Acid	HSBM	CGM
Essential	6044 (±661.2) + 0.91 (±0.129) x	6615 (±661.0) + 0.87 * (±0.111) x
Arg	676 (±94.2) + 0.88 (±0.093) x	696 (±94.2) + 0.94 (±0.205) x
His	207 (±22.7) + 0.80 (±0.118) x	223 (±22.7) + 1.21 (±0.203) x
Ile	737 (±85.1) + 0.97 (±0.123) x	804 (±85.0) + 0.77 * (±0.112) x
Leu	1121 (±135.7) + 0.92 (±0.235) x	1282 (±165.6) + 0.98 (±0.99) x
Lys	777 (±103.5) + 0.70 * (±0.094) x	836 (±103.5) + 0.09 * (±0.253) x
Met	248 (±33.5) + 1.22 (±0.311) x	289 (±33.5) + 0.55 * (±0.153) x
Phe	619 (±56.3) + 0.95 (±0.155) x	691 (±56.3) + 0.84 (±0.107) x
Thr	764 (±31.1) + 1.04 (±0.135) x	866 (±61.1) + 1.00 (±0.157) x
Val	893 (±90.2) + 0.96 (±0.125) x	966 (±90.2) + 0.81 (±0.113) x
Nonessential	7487 (±774.4) 1.30 (±0.213) x	8134 (±744.2) + 1.84 * (±0.225) x
Ala	1278 (±124.4) + 0.97 (±0.291) x	1288 (±124) + 1.58 * (±0.179) x
Asp	1537 (±154.9) + 2.07 * (±0.271) x	1629 (±158.9) + 3.86 * (±1.071) x
Glu	1934 (±237.9) + 1.43 (±0.252) x	2189 (±237.9) +3.18 * (±0.367) x
Gly	760 (±68.3) + 1.06 (±0.140) x	803 (±68.3) + 0.91 (±0.201) x
Pro	703 (±74.5) + 0.69 * (±0.214) x	784 (±74.4) + 0.92 (±0.109) x
Ser	744 (±66.5) + 0.96 (±0.132) x	812 (±66.5) + 1.25 (±0.147) x
Tyr	583 (±96.2) + 2.57 * (±0.592) x	629 (±96.2) + 4.61 * (±0.60) x
Total AA	13531 (±1426) + 1.09 (±0.164) x	14749 (±1426) + 1.25 (±0.153) x

* Means within the same column differ from total AA.

## Data Availability

Raw data database available at https://data.mendeley.com/datasets/df9zvtjmng/1. (accessed on 6 March 2022).
